# Microstructure of Haynes^®^ 282^®^ Superalloy after Vacuum Induction Melting and Investment Casting of Thin-Walled Components

**DOI:** 10.3390/ma6115016

**Published:** 2013-11-01

**Authors:** Hubert Matysiak, Malgorzata Zagorska, Joel Andersson, Alicja Balkowiec, Rafal Cygan, Marcin Rasinski, Marcin Pisarek, Mariusz Andrzejczuk, Krzysztof Kubiak, Krzysztof J. Kurzydlowski

**Affiliations:** 1 Functional Materials Research Center, Warsaw University of Technology, Woloska 141, Warsaw 02-507, Poland; 2 Faculty of Materials Science and Engineering, Warsaw University of Technology, Woloska 141, Warsaw 02-507, Poland; E-Mails: m.zagorska@inmat.pw.edu.pl (M.Z.); a.balkowiec@inmat.pw.edu.pl (A.B.); mrasin@o2.pl (M.R.); mandrzejczuk@inmat.pw.edu.pl (M.A.); kjk@inmat.pw.edu.pl (K.J.K.); 3 Guest, Keen and Nettlefolds (GKN) Aerospace Engine Systems Sweden, Trollhättan S-46181, Sweden; E-Mail: joel.andersson@gknaerospace.com; 4 Department of Engineering Science, University West, Trollhättan 46186, Sweden; 5 Department of Materials and Manufacturing Technology, Chalmers University of Technology, Gothenburg 41296, Sweden; 6 Wytwornia Sprzetu Komunikacyjnego, “Polskie Zaklady Lotnicze Rzeszow” S.A., Hetmanska 120, Rzeszow 35-078, Poland; E-Mail: rafal.cygan@wskrz.com; 7 Institute of Physical Chemistry, Polish Academy of Sciences, Kasprzaka 44/52, Warsaw 01-224, Poland; E-Mail: marcinpisarek@interia.pl; 8 Faculty of Mechanical Engineering and Aeronautics, Rzeszow University of Technology, Al. Powstancow Warszawy 8, Rzeszow 35-959, Poland; E-Mail: krkub@prz.edu.pl

**Keywords:** Haynes^®^ 282^®^, vacuum induction melting, investment casting, superalloy, microstructure

## Abstract

The aim of this work was to characterize the microstructure of the as-cast Haynes^®^ 282^®^ alloy. Observations and analyses were carried out using techniques such as X-ray diffraction (XRD), light microscopy (LM), scanning electron microscopy (SEM), transmission electron microscopy (TEM), X-ray spectroscopy (EDS), wave length dispersive X-ray spectroscopy (WDS), auger electron spectroscopy (AES) and electron energy-loss spectrometry (EELS). The phases identified in the as-cast alloy include: γ (gamma matrix), γʹ (matrix strengthening phase), (TiMoCr)C (primary carbide), TiN (primary nitride), σ (sigma-TCP phase), (TiMo)_2_SC (carbosulphide) and a lamellar constituent consisting of molybdenum and chromium rich secondary carbide phase together with γ phase. Within the dendrites the γʹ appears mostly in the form of spherical, nanometric precipitates (74 nm), while coarser (113 nm) cubic γʹ precipitates are present in the interdendritic areas. Volume fraction content of the γʹ precipitates in the dendrites and interdendritic areas are 9.6% and 8.5%, respectively. Primary nitrides metallic nitrides (MN), are homogeneously dispersed in the as-cast microstructure, while primary carbides metallic carbides (MC), preferentially precipitate in interdendritic areas. Such preference is also observed in the case of globular σ phase. Lamellar constituents characterized as secondary carbides/γ phases were together with (TiMo)_2_SC phase always observed adjacent to σ phase precipitates. Crystallographic relations were established in-between the MC, σ, secondary carbides and γ/γʹ matrix.

## 1. Introduction

Environmental demands on the next generation of aero engines may require an increased service temperature. As temperature exceeds ~650 °C, the normally-used Alloy 718 is no longer an option.

Turbine structural components are traditionally cast as single piece components, which are relatively expensive because of the low efficiency of the casting process and post cast operations. For this reason, the recent trend in fabrication of large structural components is to cast smaller parts which can be joined with rolled or forged parts. This provides the possibility to use higher strength wrought parts of relatively simple geometry with cast ones of complex geometry, which usually carry lower loads. In addition, components made of different alloys can be fabricated at a reduced cost because the parts are produced in separate processes (casting, forging, welding, *etc.*) [1]. Alloys of interest above ~650 °C temperature are, for instance; Allvac^®^ 718Plus™, Haynes^®^ 282^®^, Nimonic C263 and Waspaloy.

Here, Haynes^®^ 282^®^ is a newly developed γʹ-strengthened Ni-based superalloy, which allows for service temperature ranging from 649 to 927 °C which is significantly higher in comparison to Alloy 718 [1,2,3,4,5,6,7,8]. Such high temperature capability of Haynes^®^ 282^®^ is possible due to the specific chemical composition and its relatively low γʹ content, which also assures its thermal stability [2,3,4,5,6,7,8]. Also, this alloy features improved formability and weldability as a consequence of the lowered content of γʹ phase in comparison with, e.g., Waspaloy [9,10,11,12,13,14]. Mechanical properties are maintained at required levels despite low amounts of Al and Ti. This could possibly be explained in terms of high concentration of large atomic size alloying elements, in particular Mo and Co. On the other hand, such large atomic size elements are prone to the formation of TCP phases [15,16,17,18,19,20,21,22,23], which deteriorate mechanical properties. It is therefore important to investigate the as-cast microstructures of Haynes^®^ 282^®^, which is the aim of the present study.

## 2. Material and Experimental Procedure

### 2.1. Material for Investigation

The material used in this study, was acquisitioned from Haynes International Inc. in the form of 76 mm diameter, wrought, fully annealed bars, composition of which is given in Table 1. This table also provides information on chemical composition of the as-cast specimens.

**Table 1:** Chemical composition in wt % of Haynes^®^ 282^®^ (as received and as-cast state).

Chemical element	Chemical composition in wt % of Haynes^®^ 282^®^	
	As received	As-cast
C	0.0598	0.0563
Si	0.066	0.073
S	0.0036	0.0025
P	0.006	0.006
Mn	0.038	0.027
Cr	20.27	20.11
Mo	8.21	8.06
V	0.032	0.03
W	0.08	0.07
Ti	2.32	2.25
Co	10.02	9.95
Al	1.74	1.68
B	0.003	0.003
Nb	0.06	0.06
Ta	0.03	0.03
Mg	0.006	0.003
Fe	1.21	1.17
Zr	0.00137	0.00098
N	55 ppm	79 ppm
Ni	Balance	Balance

### 2.2. Investment Casting

**Figure 1:**
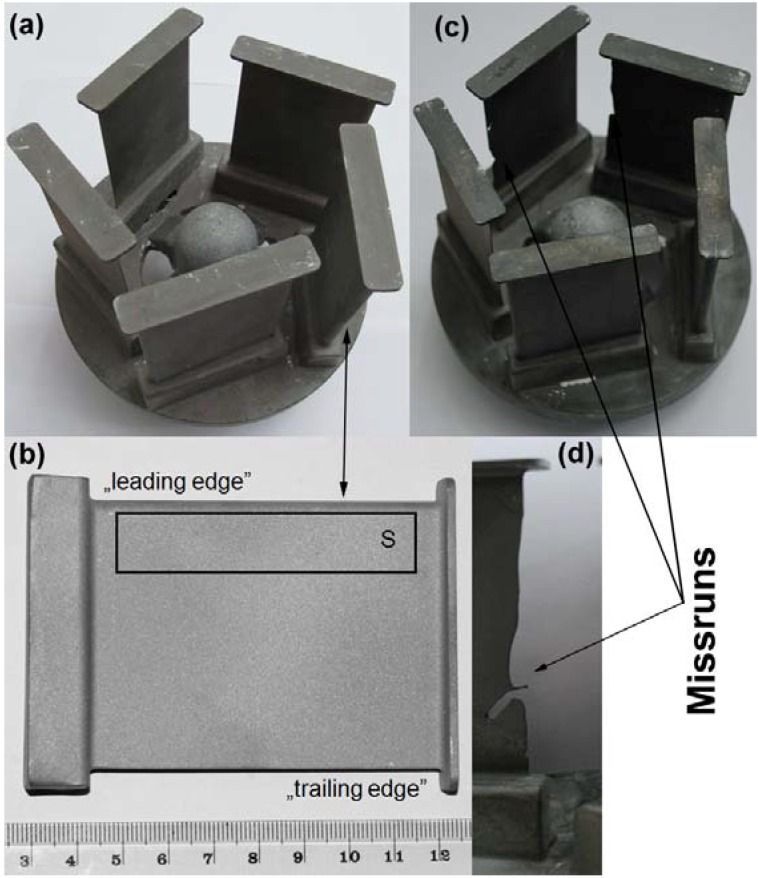
Examples of thin-walled wedges after knock-out: (**a**) and (**b**) *T*_m_ = 1550 °C; *T*_s_ = 1250 °C, where S denominates the sampling area; (**c**) and (**d**) *T*_m_ = 1550 °C; *T*_s_ = 1000 °C.

A 4.6 kg charge was melted in a Zirconia crucible in an industrial vacuum induction melting and investment casting (VIM IC) CONSARC furnace. The molten alloy was cast into a ceramic shell mould, wrapped with thermal insulating material preheated to 1250 °C (*T*_s_). The melt pouring temperature (*T*_m_) was 1550 °C. The vacuum level during melting and pouring was maintained at 2 × 10^−2^ mbar. The as-cast samples had a thin-walled “airfoil” geometry with a thickness ranging from 6.0 mm (“leading edge”) to 0.5 mm (“trailing edge”). The assembly consisted of five thin-walled cast samples, each of 0.2 kg with a height of 100 mm and a width of 70 mm—see Figure 1.

Each part was subjected to visual inspection, fluorescent penetrant inspection (FPI) and X-ray inspection by certified personnel in order to disclose any defects.

### 2.3. Microstructure Characterization of As-Cast Haynes^®^ 282^®^

Microstructure observations and analyses were carried out using the following techniques; X-ray diffraction (XRD), light microscopy (LM), scanning electron microscopy (SEM), transmission electron microscopy (TEM), high resolution scanning-transmission electron microscopy (HR-STEM), X-ray spectroscopy (EDS), wave length dispersive X-ray spectroscopy (WDS), auger electron spectroscopy (AES) and electron energy-loss spectrometry (EELS). It should be noted that due to differences in the wall thickness of the castings, samples were cut from pre-selected areas (as shown in Figure 1d). This was done for all techniques stated above.

The phase content of the as-cast, bulk and polished samples was examined by XRD using PHILIPS PW 1830 (Cu Kα radiation). The XRD patterns were acquired at a scan rate of 0.025°/s with 2θ (Bragg angle) and a scan range from 30° to 110°. Microstructure observations and analyses were carried out by LM, SEM, TEM and HR-STEM. Samples for metallographic observations were chemically etched with Kalling’s reagent. The dendritic microstructure and secondary dendrite arm spacing (SDAS) were investigated with Nikon Epiphot 220 inverted reflected LM and quantitatively analyzed using MicroMeter software.

The SEM specimens were prepared using a standard procedure for metallographic preparation. Plasma cleaning prior loading of the sample into the microscope was carried out to suppress carbon contamination during the analyses.

The structure and chemistry of the cast parts were analyzed by field emission (FE)SEM Hitachi SU70 using EDS and a WDS. For the EDS and WDS analyses a 15 kV accelerating voltage was used. The microstructure images were analyzed quantitatively in terms of the number of precipitates per area (*N_A_* parameter) and its size (*d*-equivalent diameter). The number of precipitates per volume (*N_V_* parameter) was calculated using the following equation [24]:

*N_V_* = *N_A_*/*d* (1)

Samples for TEM observations were cut from 3 mm diameter cylinders by spark erosion. Thin foils of 0.2 mm thickness were sliced by a wire saw and mechanically ground using a Gatan Dimple Grinder. Finally, thin foils were electrochemically polished by the double-jet method on a Struers device in a mixture of acetic and perchloric acids (95 and 5 vol %. respectively). The images of the microstructures were used to quantify the size and shape of γʹ precipitates. The size was quantified using the equivalent diameter *d*, defined as the diameter of a circle with the same surface area as that of the precipitate. The volume fraction (*Vv*-parameter) of gamma prime was estimated from area measurements on the TEM images, using the equation [24]:

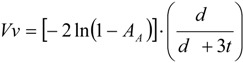
(2)
where: *A_A_* is the area fraction of gamma prime phase and *t* is the thin foil thickness.

In order to reveal the fine microstructure of precipitates, in the as-cast Haynes^®^ 282^®^, thin foils, with thicknesses lower than 100 nm were prepared for HR-STEM observations, using a single focused ion beam (FIB) system (Hitachi FB-2100) and a lift-out preparation technique.

Microstructure investigations were also performed using a Jeol JEM 1200EX II (operating at 120 kV) and HR-STEM Hitachi HD-2700 equipped with EDS, EELS operating at 200 kV. This microscope has a high angle annular dark field (HAADF) detector, with a collecting angle from 70 to 370 mrad, which enables imaging with compositional contrast.

An Auger microprobe analyzer, Microlab 350 (Thermo Electron), where the AES has a lateral resolution of about 20 nm was used to disclose whether any boron and/or carbon were present in certain phase constituents. An Avantage-based data system was used for data acquisition and processing.

## 3. Results and Discussion

### 3.1. Investment Casts

The thin-walled “wedge” castings fabricated and examined in this study are shown in Figure 1. The investment casting temperatures were *T*_s_ = 1250 °C and *T*_m_ = 1550 °C for the mould and alloy, respectively. FPI and X-ray inspections revealed no critical defects in the castings such as; miss runs, hot tears, porosity, dross or non-metallic inclusions.

Relatively high *T*_s_ and *T*_m_ temperatures were utilized during the casting process to obtain satisfactory filling of the thin-walled sections of the patterns. It should be noted that lower temperatures would be used for typical industrial applications, e.g., *T*_s_ = 1000 °C and *T*_m_ = 1500–1550 °C. These temperatures were also tested in this project, however, miss runs were observed in the “trailing edges” of the casting, as shown in Figure 1c,d.

### 3.2. Microstructure of As-Cast Haynes^®^ 282^®^

The chemical composition of the as-cast Haynes^®^ 282^®^ given in Table 1 reveals that the VIM IC process does not significantly change the chemical composition of the alloy. The observed marginal differences are the effect of segregation and evaporation of certain alloying elements, which is typical for casting as a process.

**Figure 2:**
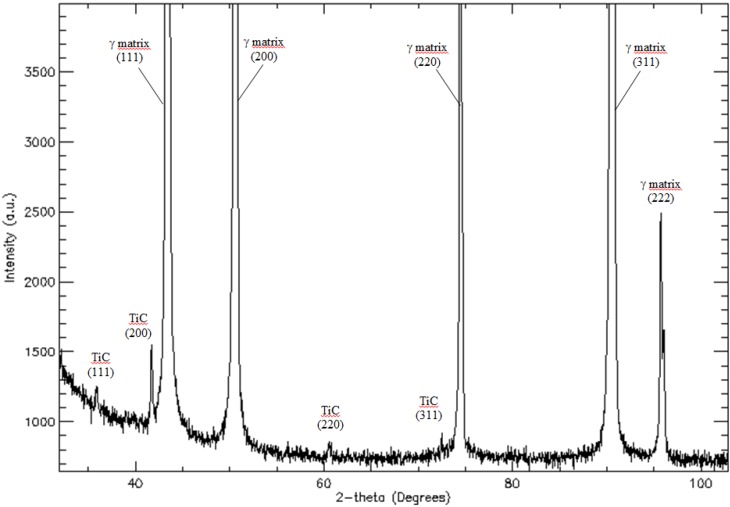
X-ray diffraction (XRD) patterns as obtained for the as-cast Haynes^®^ 282^®^.

The XRD pattern for as-cast Haynes^®^ 282^®^ is shown in Figure 2 and summarized in Table 2. The pattern obtained, clearly reveals peaks of two phases: γ matrix and TiC phases. In Table 2, the values of the *d*_(*hkl*)_ spacing and a_0_ lattice parameter are given as measured on the XRD patterns. These values agree well with reference powder diffraction data both for TiC and γ(Ni, Fe) austenite.

**Figure 3:**
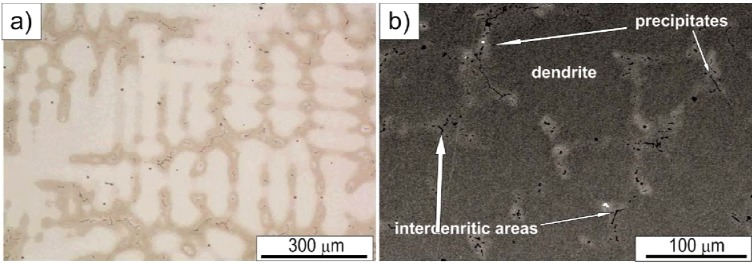
Dendritic microstructure of the as-cast Haynes^®^ 282^®^: (**a**) Light Microscopy (LM) image; (**b**) Scaning Electron Microscopy in backscattered electrons mode (SEM-BSE) image.

Microstructures representative of as-cast Haynes^®^ 282^®^ are shown in Figure 3. As-cast samples reveal heterogeneous dendritic structure with various precipitates. A strong micro-segregation of alloying elements (Mo, Ti and Cr—confirmed by EDS analyses) is clearly seen on the etched samples due to a difference in contrast between dendrites and inter-dendritic areas. Quantitative image analysis of the as-cast microstructure revealed that the average secondary denrite arm spacing (SDAS) is in the range of 46–55 μm. The LM image reveals that large gray irregular carbides preferentially precipitated in inter-dendritic areas. On the other hand, nitrides are homogeneously dispersed in the as-cast microstructure (nitrides are easily identified due to their regular, angular shapes and orange-red color). SEM images, in Figure 3b, reveal additional precipitates, apart from MC and MN, clustered in the inter-dendritic areas. Based on their mass contrast and chemical composition, these precipitates/particles were classified into four specific groups as described in more detail below.

**Table 2:** The measured values of *d*_(*hkl*)_ spacing and a_0_ lattice parameters for γ, γʹ, metallic carbides (MC) and secondary carbides phases in the as-cast Haynes^®^ 282^®^.

(*hkl*)	*d_(hkl)_* [Å]	*a*_0_ [Å]	*d_(hkl)_* [Å]	*a*_0_ [Å]
	Measurement: TEM *; XRD **		Powder diffraction file	
γ matrix FCC Fm3m (225)			PDF No. 47-1417	
(111)	2.08 *; 2.08 **	3.61 *; 3.61 **	2.079	3.601
(200)	1.79 *; 1.80 **	3.58 *; 3.61 **	1.800	3.600
(311)	1.08 *; 1.09 **	3.57 *; 3.60 **	1.085	3.599
(222)	1.04 *; 1.04 **	3.60 *; 3.60 **	1.038	3.596
(400)	0.89 *; – **	3.56*; – **	0.900	3.600
(331)	0.83 *; – **	3.61*; – **	0.826	3.601
**γʹ FCC ordered L1_2_**			**PDF No. 09-0097**	
(100)	3.57 *; – **	3.57 *; – **	3.600	3.600
(111)	2.08 *; – **	3.61 *; – **	2.074	3.593
(200)	1.79 *; – **	3.58 *; – **	1.799	3.598
(211)	1.47 *; – **	3.60 *; – **	1.461	3.579
(311)	1.08 *; – **	3.57 *; – **	1.078	3.575
(222)	1.04 *; 1.02 **	3.60 *; 3.53 **	1.032	3.575
(400)	0.89 *; – **	3.56 *; – **	0.893	3.572
(331)	0.83 *; – **	3.61 *; – **	0.819	3.570
**MC (TiC) FCC Fm3m (225)**			**PDF No. 32-1383**	
(111)	2.46 *; 2.50 **	4.26 *; 4.33 **	2.499	4.328
(200)	2.14 *; 2.17 **	4.28 *; 4.17 **	2.164	4.164
(220)	1.49 *; 1.53 **	4.23 *; 4.32 **	1.530	4.328
(311)	1.29 *; 1.30 **	4.28 *; 4.32 **	1.305	4.327
**Secondary carbides: M_23_C_6_ FCC Fm3m (225)/M_6_C FCC Fd3m (227)**			**PDF No. 85-1281/PDF No. 47-1192**	
(111)	6.19 *; – **	10.72 *; – **	6.154 /6.429	10.659 /11.130
(200)	5.40 *; – **	10.79 *; – **	5.330 /5.569	10.659 /11.130
(220)	3.79 *; – **	10.71 *; – **	3.769 /3.939	10.660 /11.130
(311)	3.30 *; – **	10.96 *; – **	3.214 /3.359	10.660 /11.130

Notes: * indicates the results obtained with electron diffraction and ** with X-ray diffraction.

**Figure 4:**
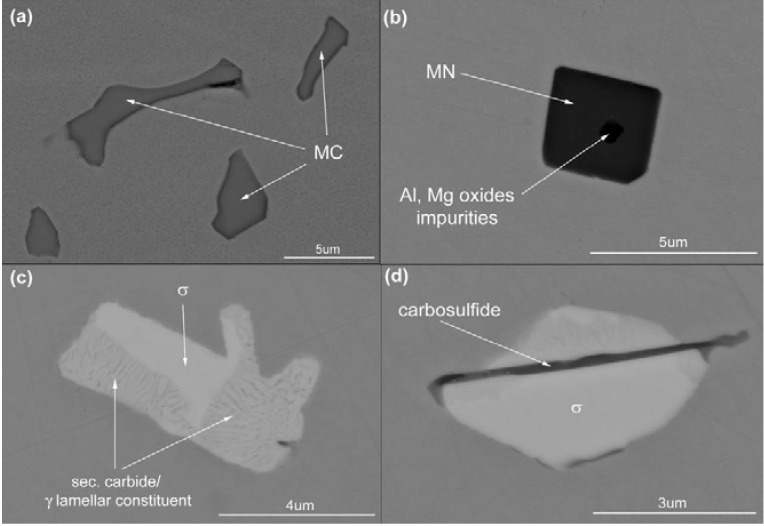
Representative precipitates in the as-cast Haynes^®^ 282^®^: (**a**) metallic carbides, MC; (**b**) metallic nitrides, MN, and oxide impurities; (**c**) σ phase and secondary carbide/ γ lamellar constituent; (**d**) M_2_SC and σ phase.

MC precipitates, appear in large number (see Table 3) as “gray” particles. These precipitates are concentrated in the interdendritic areas. Representative MC particles are shown at high magnification in Figure 4a (Please note that the Z-contrast is the same for all these precipitates). Semi-quantitative EDS analyses of MC precipitates revealed approximately 50 at % of C, 30 at % of Ti, 14 at % of Mo and 2 at % of Cr (see Table 4). The last three elements, Ti, Mo and Cr, are carbide forming elements [25,26,27,28].

**Table 3:** Size and distribution frequency of secondary phases in the as-cast Haynes^®^ 282^®^ alloy (metallic carbides (MC), metallic nitrides (MN)).

Precipitation	MN	MC	σ and lamellar constituent	M_2_SC	γʹ dendrite	γʹ interdendritic
*d* [μm]	4.04	2.48	2.87	0.76	0.074	0.113
*N_A_* [L/mm^2^]	55	550	19	6	–	–
*N_V_* [L/mm^3^]	14 × 10^3^	222 × 10^3^	7 × 10^3^	8 × 10^3^	–	–
*V_V_* [%]	–	–	–	–	9.6	8.5

**Figure 5:**
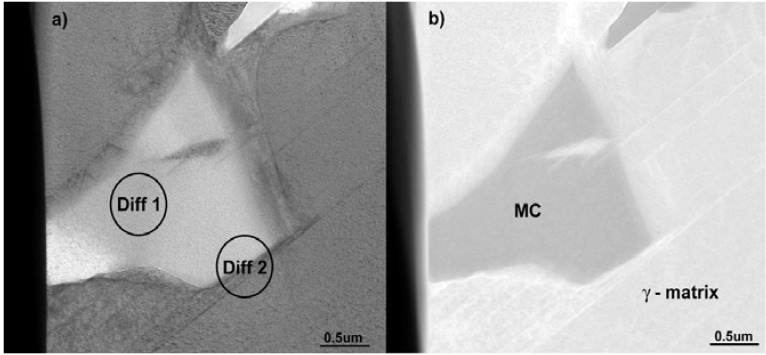
Microstructure of a MC: (**a**) Scanning Transmission Electron Microscpy in Bright Field (BF-STEM); and (**b**) Scanning Transmission Electron Microscpy in High-Angle Annular Dark Field (HAADF-STEM).

**Figure 6:**
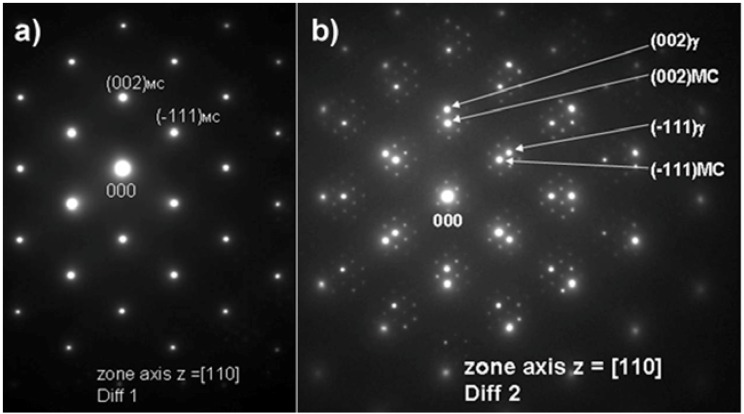
Selected area electron diffraction patterns for the MC phase constituent shown in (**a**) together with superimposed Moiré electron diffraction pattern for MC and γ phase in (**b**).

The MC particles were also analyzed using TEM. Bright field (BF) and high angle annular dark field (HAADF) images of these particles are shown with corresponding electron diffraction patterns in Figure 5 and Figure 6, respectively. It should be noted that the pattern shown in Figure 6a clearly reveals reflections from a FCC crystal structure. The *d*_(*hkl*)_-spacing and a_0_ lattice parameters measured on the basis of electron diffraction patterns, listed in Table 2, are in good agreement with the theoretical values.

**Table 4:** Chemical composition in at % of MC, MN, σ and secondary carbide/γ lamellar constituent as obtained through X-ray spectroscopy (EDS) analyses.

Phase	Chemical composition in at %			
	MC	MN	σ	secondary carbide/γ lamellar constituent
N	–	56.7 ± 0.5	–	–
C	52.7 ± 2.4	–	–	20.7 ± 7.9
Si	–	–	1.0 ± 0.4	0.7 ± 0.1
Ti	30.7 ± 1.8	42.5 ± 0.9	1.0 ± 0.3	–
Cr	1.7 ± 0.5	0.3 ± 0.1	29.0 ± 0.7	22.4 ± 3.4
Co	–	–	9.5 ± 0.7	8.0 ± 1.3
Ni	1.4 ± 0.7	0.7 ± 0.1	27.8 ± 0.8	23.7 ± 6.0
Mo	13.6 ± 0.8	–	31.0 ± 0.6	24.1 ± 0.7
Al	MC	MN	0.5 ± 0.1	–

Based on the electron diffraction patterns (Figure 6b) the following, typical cube-cube orientation relationships between the MC and γ-matrix were confirmed:

[110]MC//[110]γ
(3)

(–111)MC//(–111)γ
(4)

It should be noted that MC carbides are important constituents in Ni based superalloys. Formation of primary carbides begins in the melt due to segregation of carbon, which reacts with elements such as Ti, Mo, Cr and Nb. Fine MC precipitates at grain boundaries or in the matrix which strengthens the alloy and also ties-up some elements that would otherwise promote phase instability during heat treatment and service. These carbides may transform into M_23_C_6_ and/or M_6_C at 760–980 °C and 815–980 °C, respectively, which are rich in Cr, Mo and/or W [7,26]. The most common transformation can be described as:

MC + γ = M_23_C_6_ and/or M_6_C + γʹ (5)

**Figure 7:**
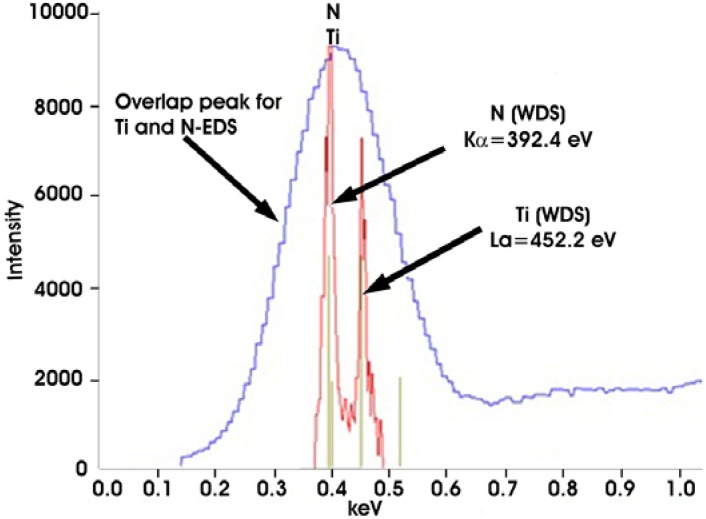
EDS and wave length dispersive X-ray spectroscopy (WDS) patterns for MN.

The homogenously distributed precipitates exemplified in Figure 4b are characterized as TiN. These particles have significantly lower number density in comparison with MC—see Table 3. SEM images of these precipitates (Figure 4b) exhibit homogenous Z-contrast with a dark nucleus centre of regular shape. Results from the measurement of their chemical analyses are given in Table 4. Since the spectra show a strong peak in the region of overlapping lines of Ti and N, additional WDS analyses were conducted (Figure 7) with the conclusion that these are TiN precipitates which confirms the findings using LM (orange-red color) [26].

The EDS chemical analyses also revealed strong signals of Mg, Al and O at the centre of the TiN precipitates. This suggests that nitrides nucleate around non-metallic oxide impurities. It should be noted that TiN particles are not influenced by the potential heat treatments of the casting and are insoluble up to the melting point. At their standard concentration in superalloys, they generally have little influence on the mechanical properties [26].

The “white phase” particles are present in the interdendritic areas. The number density of these particles is much lower than the MC carbides, see Table 3. Detailed SEM examinations (Figure 4c) have shown that they often constitute: (a) compact-precipitation with homogenous Z-contrast and (b) lamellar appearance. The results of the EDS analyses for both regions are listed in Table 4 above. The compact phase contains equal amounts (30 at %) of Cr, Ni and Mo and 10 at % of Co. The chemical composition of the lamellar constituent areas are similar to that of the compact phase, however, with a significant concentration of C. As the spatial resolution of the EDS method is relatively low, FIB samples were cut-out from the compact precipitation and lamellar constituent regions and were investigated by AES and TEM analysis methods.

**Figure 8:**
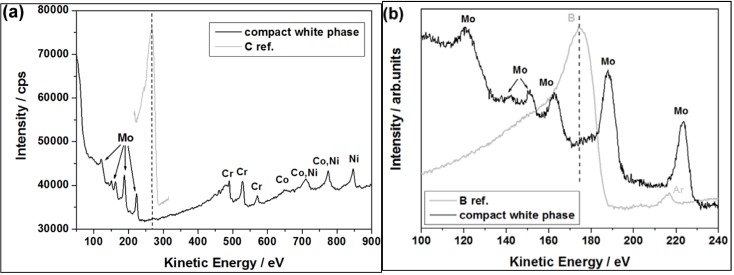
Local auger electron spectroscopy (AES) spectra for the compact “white phase” (**a**) with reference carbon peak; and (**b**) with reference boron peak.

Since elements like Mo and Cr are generally known to have a high affinity for boron/carbon and the solid solubility of boron/carbon in the γ phase is very low, it is likely that borides (like M_3_B_2_ and/or M_5_B_3_) and/or secondary carbides (like M_23_C_6_ and/or M_6_C) easily form. This is commonly observed in many Ni based superalloys [29,30]. Thus, AES was used to analyze light elements (C and B) in the “white phase”. A representative local auger spectrum for the compact “white phase” is shown in Figure 8. The obtained results confirm that the phase is rich in Mo, however boron as well as carbon was not detected. It suggests that the compact “white phase” should be one of the TCP phases, and not a boride or carbide.

**Figure 9:**
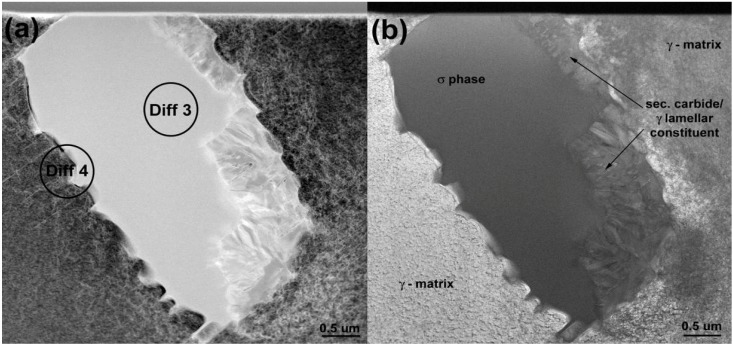
Microstructure of σ phase particles: (**a**) HAADF-STEM and (**b**) BF-STEM.

**Figure 10:**
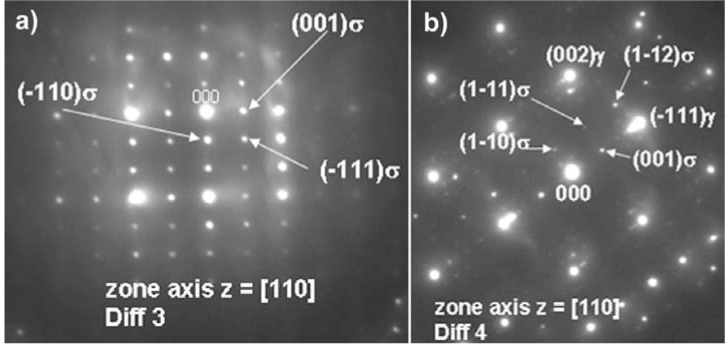
Diffraction pattern of the sigma phase particle shown in Figure 9a.

A TEM micrograph of a TCP particle is shown in Figure 9 with corresponding electron diffraction pattern in Figure 10. This pattern clearly reveal reflections from a tetragonal, P4_2_/mnm crystal structure with a = 8.7 Å and c = 4.7 Å. The measured values of the d_(hkl)_ spacing, a_0_ and c_0_ lattice parameters, are listed in Table 5 together with powder diffraction data for σ phase of various chemical compositions. A good agreement between the reference and experimental data obtained in this study can be noted. Furthermore, the current data reveal an orientation relationship in between the TCP phase precipitates and the γ matrix which has been already reported in [16,22]:

[110]σ//[110]γ
(6)

(001)σ//(–111)γ
(7)

The intermetallic σ phase is hard and brittle. It is detrimental to the mechanical properties of superalloys if present in the form of elongated particles or as grain boundary films. On the other hand, precipitates that are small and spherical in shape may improve creep resistance. Nevertheless, the formation of the σ phase is always a concern as it depletes refractory metals in the γ matrix, causing reduction in strength [15,16,17,18,19,20,21,22,26].

**Table 5:** The measured values of d_(hkl)_-spacing and lattice parameters for σ phase in the as-cast Haynes^®^ 282^®^ superalloy.

(*hkl*)	*d_(hkl)_* [Å]	*a*_0_ [Å]	*c*_0_ [Å]	*a*_0_ [Å]	*c*_0_ [Å]
	Measurement			Powder diffraction data	
σ phase-Tetragonal, P4_2_/mnm (136)					
(110)	6.14	–	–	–	–
(001)	4.66	–	–	–	–
(111)	3.73	–	–	9.170 *	4.741 *
(221)	2.57	–	–	8.810 **	4.560 **
(112)	2.20	8.68	4.66	8.790 ***	4.544 ***

Notes: * σ-Fe-Cr-Mo (PDF-09-0050); ** σ-Cr-Co (PDF-09-0052); *** σ-Fe-Cr (PDF-05-0708).

**Figure 11:**
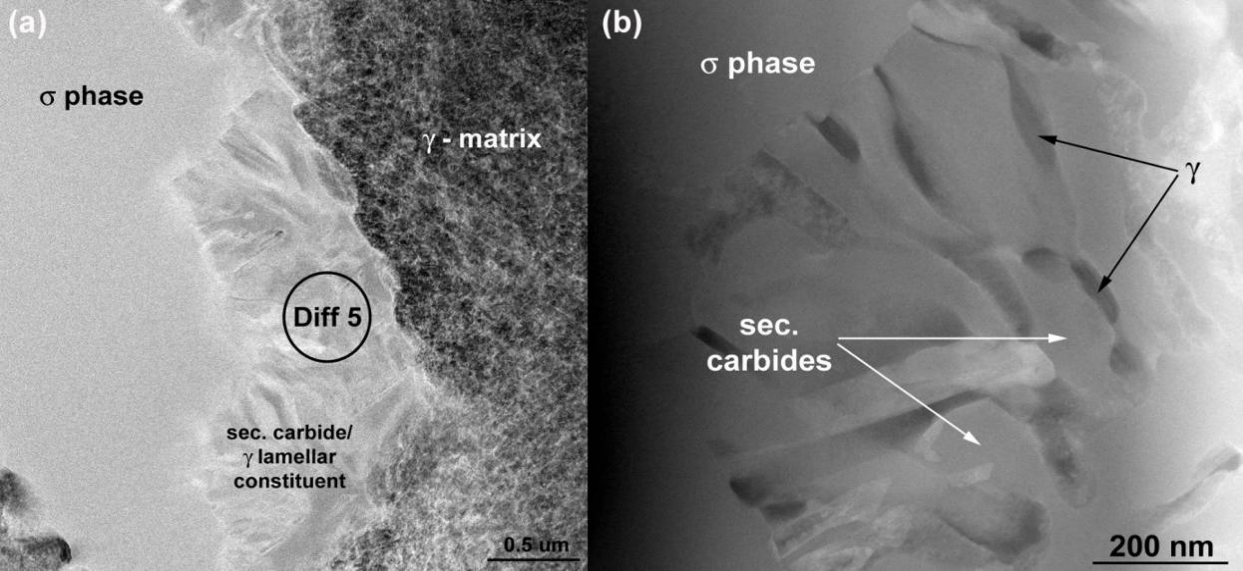
Microstructure of lamellar secondary carbide/γ constituent (HAADF-STEM).

**Figure 12:**
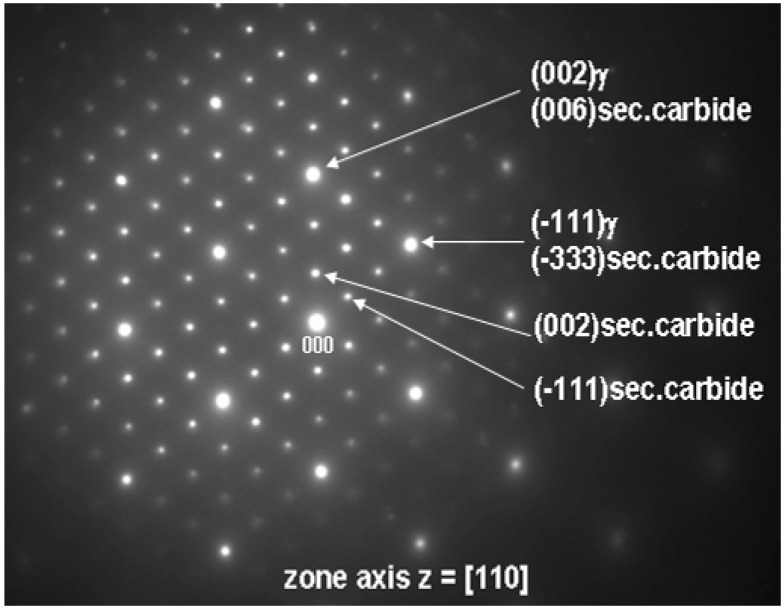
Selected area electron diffraction patterns for lamellar secondary carbide/γ obtained from regions shown in Figure 11a.

TEM was also used to investigate the lamellar areas, exemplified in Figure 11. The corresponding electron diffraction pattern (Figure 12) demonstrates that both phases possess FCC crystal structure. Reflections from the “bright phase” are present at every third gray phase dot and the following, typical cube-cube orientation relationships were confirmed:

[110] sec.carbide//[110] γ
(8)

(−111) sec.carbide//(−111) γ
(9)

**Figure 13:**
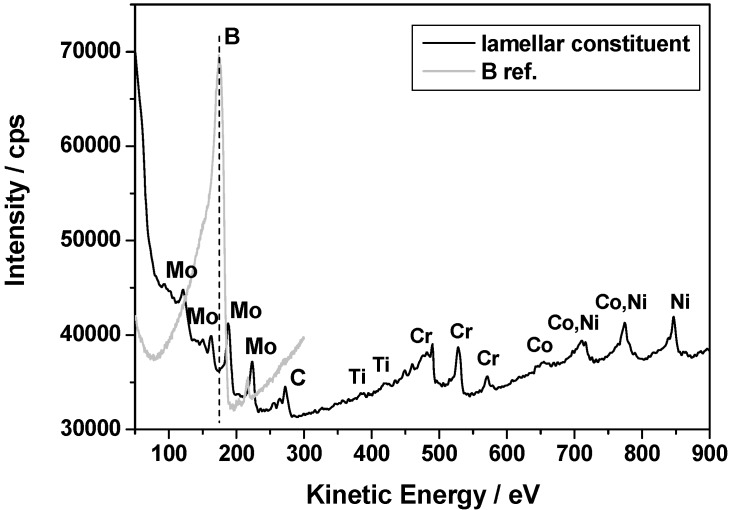
Local AES spectra for “bright phase” in lamellar constituent with reference boron peak.

Such relationships are characteristic of secondary (M_23_C_6_ and/or M_6_C) carbides and γ matrix in cast superalloys [18,25]. The values of *d*_(*hkl*)_ spacing and a_0_ lattice parameters estimated from the diffraction patterns are listed in Table 5 together with reference powder diffraction data for Cr_23_C_6_ and Mo_3_Fe_3_C carbides. Best fit was achieved for M_23_C_6_ carbide, however M_6_C is also probable. EDS measurements (Table 4) indicate that the “bright phase” located within the lamellar constituent is rich in Mo, however significant solubilities for Cr, Ni, Co and C have been also observed. Local AES spectra, as shown in Figure 13, confirm the results obtained by the EDS technique. It should be noticed that boron was not detected. It is reasonable to believe that the boron content in the alloy is too low to form any borides in the as-cast state which suggests that B is soluble in the matrix. Boron may reduce solubility of carbon in the γ matrix, which promote precipitation of carbides [26]. From this part of the study it can therefore be concluded that the “white phase” constitutes a TCP σ phase together with a lamellar constituent consisting of γ and secondary carbide. To the authors’ knowledge, these type of objects have not previously been reported for in the Haynes^®^ 282^®^ superalloy.

**Figure 14:**
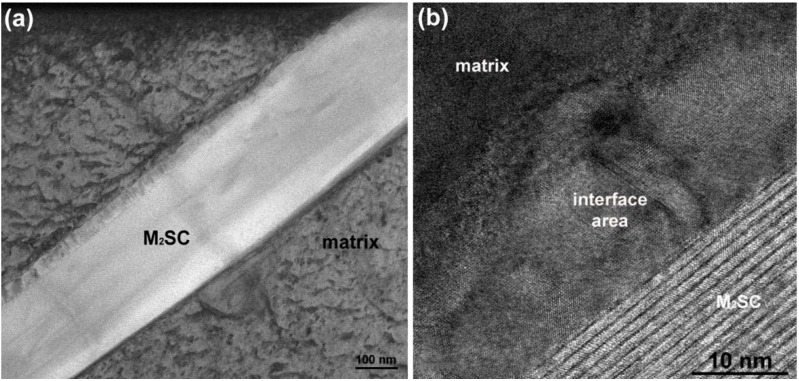
Microstructure of a M_2_SC carbosulphide: (**a**) BF-STEM; and (**b**) high resolution image.

The “black needles/platelets” constituents, which have been analyzed in this study, are observed adjacent to σ phase precipitates (see Figure 4d). Their density is relatively low, see Table 3. HR-STEM micrographs of a “needle” shown in Figure 14 reveals so called “nano-laminates” within the microstructure being typical for MAX phases (M*_n_*_+1_AX*_n_*, where *n* = 1, 2 or 3, M is an early transition metal, A is an A-group element, and X is either C or N) [31].

**Figure 15:**
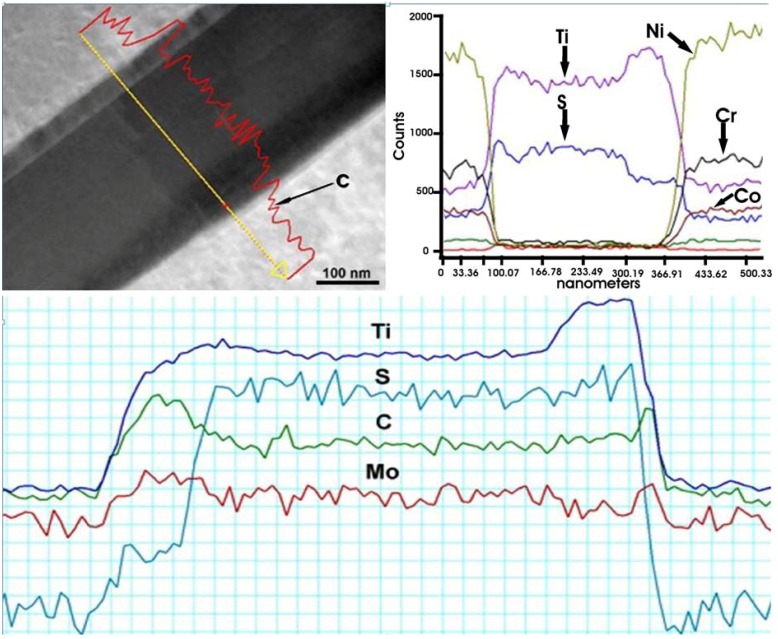
EDS and electron energy-loss spectrometry (EELS) line-scans of elements in M_2_SC carbosulfide.

The EDS and EELS spectra (Figure 15) reveal that the “needles” contain S and Ti. Some concentration of C (especially on the interface) and Mo were also detected. Sulfur is known to be a harmful element in superalloys. It may segregate to the grain boundaries [26,27,28,30] increasing the susceptibility towards embrittlement (intergranular fracture). Sulfur also reduces ductility through the formation of sulfide/carbosulfide particles with needle/plate morphology (mostly with titanium) [26] of M_2_SC stoichiometry (where M = Ti, Mo, Nb, Cr, and Fe). The distance in between the “nano-laminates” measured in this study is 11.17 Å, which is in a good agreement with the theoretical value of c_0_ lattice constant for Ti_2_SC. It should be noted that Ti_2_SC has a hexagonal unit cell (P6_3_/mmc space group) with lattice constants of *a*_0_ = 3.22 Å and *c*_0_ = 11.22 Å [32].

**Figure 16:**
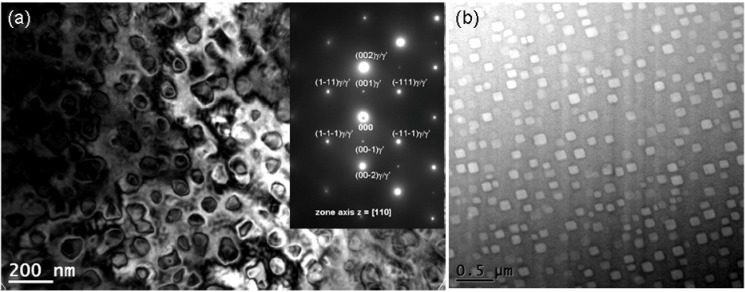
A BF TEM image and selected area electron diffraction pattern for γ/γʹ type microstructure in the as-cast Haynes^®^ 282^®^ superalloy: (**a**) dendrite; and (**b**) interdendritic area.

A bright-field TEM image of the matrix nanostructure of the as-cast Haynes^®^ 282^®^ alloy is shown in Figure 16 with the corresponding electron diffraction pattern. Within the dendrites, the nano particles appears mostly in the form of spherical, fine precipitates (74 nm), while coarser (113 nm), cubic precipitates are present in the interdendritic areas. The volume content of the precipitates within the dendrites and interdendritic areas is 9.6% and 8.5%, respectively. The electron diffraction pattern in Figure 16 reveals reflections from two phases; FCC ordered L1_2_ and FCC A1. The estimates of *d*_(*hkl*)_ spacing and a_0_ lattice parameters are listed in Table 2 together with the powder diffraction reference data for γʹ-Ni_3_Al and γ-(Ni,Fe) austenite. The agreement between these data suggests that these precipitates are nickel aluminides. Such a conclusion is confirmed by EDS analyses for the γʹ phase, which primarily revealed Ni, Al and Ti. It is thus concluded that the FCC A1 matrix (γ phase Ni-Cr-Co-Mo solid solution) of as-cast Haynes^®^ 282^®^ alloy is precipitation strengthened by the FCC ordered L1_2_, coherent Ni_3_(Al,Ti) γʹ phase. These phases remain in the following crystallographic orientation relationship:

[110]γʹ//[110]γ
(10)

(–111)γʹ//(–111)γ
(11)

In the as-cast state of Ni-based superalloys which contain a high number and concentration of alloying elements, variation in the nature (size, shape and volume content) of γʹ precipitates is commonly observed due to the effects of dendritic microsegregation and local cooling rates during cast solidification. This results in the formation of finer γʹ precipitates in the dendrites and coarser γʹ precipitates in the interdendritic regions [33].

### 3.3. Solidification of Haynes^®^ 282^®^ Superalloy

TCP phases are intermetallic compounds, generally occurring in a plate-like morphology [34]. They form when the ratio of elements such as Cr, Co and Ni exceed certain levels, dependent on the exact system involved [35]. As the level of refractory elements (e.g., Mo) exceed the solubility limit in the FCC matrix, the TCP phase will form; *i.e.*, γ → σ [34]. The σ phase, as pointed out in this case, is most often found to have a degrading impact on mechanical properties since it depletes the matrix from strengthening refractory metals (*i.e*., Mo) and chromium from the γ phase matrix [36]. It has been shown by others as well as in the present study that the σ phase is strongly enriched in Mo and slightly enriched in Cr [37]. Eutectic transformations which sometimes are encountered in solidification of superalloys are governed by an invariant reaction which involves a liquid phase being in equilibrium with other phases at a precise temperature and composition, resulting in competitive growth [38]. The final eutectic microstructure usually appears as lamellae aligned parallel to the direction of heat extraction. However, the morphology may be altered to non-competitive growth depending on the boundary conditions. Eutectic colonies are usually formed as a consequence of constitutional undercooling resulting from rejection of impurity elements, the growth rate, and thermal gradients at the solid/liquid interface [39]. The precipitates of σ phase in superalloys have most often been observed to precipitate through solid state process from the γ matrix and generally reveal plate-like morphology [40] and may also act as nucleation sites for other types of TCP phases [41]. Almost no studies can be found on how σ phase is formed during casting and how it transforms during heat treatment in superalloys.

#### 3.3.1. Solidification Characteristics

**Figure 17:**
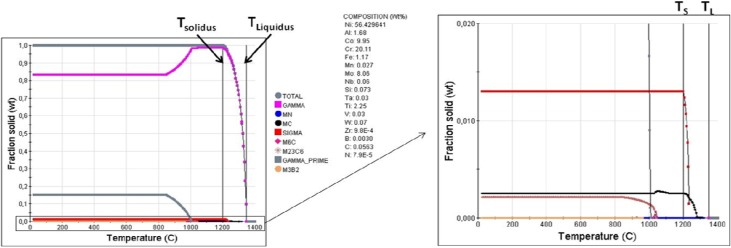
Calculated solidification sequence in the Haynes^®^ 282^®^.

The as-cast microstructure exemplified in Figure 4 and Figure 14 prove the presence of γ, γʹ, TiN, (TiMoCr)C, σ phase, M_2_SC and secondary carbide (M_6_C and/or M_23_C_6_). It can be seen that a blocky phase constituent exists adjacent to a lamellar area. Chemical analyses reveal that the blocky phase, Figure 4c, had a high content of Cr, Mo (B and C were not detected), and was characterized as σ phase using TEM. JMatPro software [42], version 7.0, using its Ni-base database was utilized to simulate the solidification sequence. It should be noted that this version of JMatPro does not include S, P or Mg in the Ni-database, hence no M_2_SC can be predicted. Scheil’s equation is incorporated in this software which considers segregation of alloying elements during solidification. The results of this exercise are shown in Figure 17 and Table 6 which indicates the following solidification sequence:

L → Lʹ + γ → Lʹʹ + γ + MN → Lʹʹʹ + γ + MN + MC → Lʹʹʹʹ + γ + MN + MC+ σ → γ + MN + MC + σ + M_6_C + M_23_C_6_ + γʹ + M_3_B_2_ (12)

**Table 6:** Calculated nucleation temperature for Haynes^®^ 282^®^.

Start nucleation temperature [°C]	**T_Liquidus_**	**γ**	**MN**	**MC**	**σ**	**T_Solidus_**	**M_6_C**	**M_23_C_6_**	**γ'**	**M_3_B_2_**
	1345	1345	1315	1310	1235	1200	1046	1040	1010	960

In the simulation, γ and σ phase formed at the end of simulation. Thus, it seems reasonable that secondary carbides such as M_6_C and/or M_23_C_6_ should form below the solidus temperature as it normally forms through decomposition of MC. However, the blocky phase constituent as can be visualized in Figure 4 was characterized as σ phase and not MC phase. The solidification process results in microsegregation between the dendrite core and interdendritic region. Increased Mo content during the solidification promotes the formation of σ phase which hypothetically later could decompose to secondary carbides such as M_23_C_6_ or M_6_C through solid state transformation *i.e.*, a eutectoid reaction (σ + C (insoluble in the γ phase) → γ + M_23_C_6_ or M_6_C) since the solubility of C dramatically decreases within the γ phase at lower temperature. Hypothetically what can be assumed is that solidification starts with formation of γ phase wherein TiN starts to nucleate followed by non-associated (TiMoCr)C and later σ + γ phase as a final reaction. As temperature decreases below the solidus temperature, the solubility of C within the γ phase drastically decreases which in turn promotes decomposition of σ phase (+C insoluble in the γ phase) through solid state reaction forming γ + M_23_C_6_ or a M_6_C eutectoid constituent followed by the γʹ phase:

L → Lʹ + γ → Lʹʹ + γ + TiN → Lʹʹʹ + γ + TiN + (TiMoCr)C + (TiMo)_2_SC → Lʹʹʹʹ + TiN + γ + TiMoC + (TiMo)_2_SC + σ → γ + TiN + TiMoC + (TiMo)_2_SC + σ + (M_23_C_6_ or M_6_C) + γ' (13)

Since the JMatPro software does not include S, P or Mg in the Ni-database, no M_2_SC can be predicted. It can only be hypothetically assumed that carbosulfide precipitation takes place based on the microstructural observations. It is a common fact that sulfur is almost insoluble in γ matrix but is soluble in MC, thus as a result, we observed formation of M_2_SC carbosulfides [32]. It must be also noted that the investigated needle shaped M_2_SC constituents usually were observed adjacent to σ phase precipitates. This suggests that the M_2_SC phase may precipitate prior to σ phase and can act as nucleation sites for the σ precipitation. A similar phenomenon was observed during solidification of alloy 718 where the needle like M_2_SC precipitated prior to Laves phase and acted as nucleation sites for this phase [32].

## 4. Conclusions

Cast parts made of Haynes^®^ 282^®^ exhibit complex dendritic microstructure with the following phases frequently observed: γʹ, carbides (MC), nitrides (MN), carbosulphides (M_2_SC) phase precipitates.

Important features of the microstructure are: σ phase and lamellar (secondary carbide and γ eutectoid). To the authors’ knowledge, these type of phases have not been reported for the Haynes^®^ 282^®^ superalloy previously.

The microstructure of as-cast Haynes^®^ 282^®^ alloy has been quantified in terms of the volume fraction of each precipitate observed in the γ phase matrix. This paper presents a comprehensive description of the microstructure of parts made of Haynes^®^ 282^®^ alloy by investment casting, which sets the stage for optimizing their post-casting heat treatment.

Based on the detailed microstructure observations described in the present study, it can be concluded that:

- The FCC A1 matrix (γ- Ni-Cr-Co-Mo based solid solution) is precipitation strengthened by coherent, ordered Ni_3_(Al,Ti) γʹ phase (FCC ordered L1_2_ crystal structure). Variation in the nature (size, shape and volume content) of γʹ precipitates is observed due to the effects of microsegregation and local cooling rates during cast solidification.
- Primary carbides MC (where M = Ti, Mo and Cr), of irregular shape and TiC-like FCC B1 crystal structure, are preferentially precipitated in the interdendritic areas.
- Primary nitrides MN, identified as TiN, are homogeneously dispersed in the entire volume.
- The σ phase precipitates have tetragonal P42/mnm crystal structure and globular form. They are preferentially precipitated in the interdendritic areas. They contain 30 at % Cr, Ni and Mo and 10 at % Co (small concentrations of Ti, Si and Al were also detected).
- Lamellar eutectoid and carbosulfide phases were always observed in close proximity to the σ phase precipitates. The Lamellar eutectoid constituent consists of: (a) chromium/molybdenum-rich secondary carbide and (b) γ phase. Carbosulfides are described by the general formula M_2_SC (M = Ti and Mo).
- The phases identified in the samples revealed the following crystallographic orientation relationships:
[110]MC//[110] sec.carbide//[110]σ//[110]γʹ//[110]γ
(14)
(−111)MC//(−111) sec.carbide//(001)σ//(−111)γʹ//(−111)γ
(15)
